# Quality of Antenatal Care for Women Who Experience Imprisonment in Ontario, Canada

**DOI:** 10.1001/jamanetworkopen.2020.12576

**Published:** 2020-08-06

**Authors:** Alison Carter Ramirez, Jessica Liauw, Alice Cavanagh, Dustin Costescu, Laura Holder, Hong Lu, Fiona G. Kouyoumdjian

**Affiliations:** 1 Department of Obstetrics and Gynecology, McMaster University, Hamilton, Ontario, Canada; 2 Department of Obstetrics and Gynecology, University of British Columbia, Vancouver, British Columbia, Canada; 3 Michael G. DeGroote School of Medicine, McMaster University, Hamilton, Ontario, Canada; 4 Faculty of Health Sciences, McMaster University, Hamilton, Ontario, Canada; 5 ICES, Toronto, Ontario, Canada; 6 Department of Family Medicine, McMaster University, Hamilton, Ontario, Canada

## Abstract

**Importance:**

Women who experience imprisonment have high morbidity and an increased risk of adverse pregnancy outcomes. Antenatal care could modify pregnancy-related risks, but there is a lack of evidence regarding antenatal care in this population.

**Objectives:**

To examine antenatal care quality indicators for women who experience imprisonment and to compare these data with data for the general population.

**Design, Setting, and Participants:**

This population-based, retrospective cohort study used linked correctional and health administrative data from women released from provincial prison in Ontario, Canada, in 2010 and women in the general population with deliveries at 20 weeks’ gestation or greater from January 1, 2005, to December 31, 2015. Data analysis was performed from January 1, 2017, to May 4, 2020.

**Exposures:**

Pregnancies in women with time in prison during pregnancy (prison pregnancies), pregnancies in women with time in prison but not while pregnant (prison control pregnancies), and pregnancies in women in the general population (general population pregnancies).

**Main Outcomes and Measures:**

Antenatal care quality indicators: first-trimester visit, first-trimester ultrasonography, and 8 or more antenatal care visits.

**Results:**

A total of 626 prison pregnancies in 529 women (mean [SD] age, 26.6 [5.4] years), 2327 prison control pregnancies in 1570 women (mean [SD] age, 26.2 [5.4] years), and 1 308 879 general population pregnancies in 884 063 women (mean [SD] age, 30.3 [5.3] years) were studied. Of 626 prison pregnancies, 193 women (30.8%; 95% CI, 27.1%-34.6%) had a first-trimester visit, 272 (48.4%; 95% CI, 44.4%-52.4%) had at least 8 antenatal care visits, and 209 (34.6%; 95% CI, 31.0%-38.4%) received first-trimester ultrasonography. In 2327 prison control pregnancies, 1106 women (47.5%; 95% CI, 45.3%-49.8%) had a first-trimester visit, 1356 (59.2%; 95% CI, 56.9%-61.4%) had 8 or more antenatal care visits, and 893 (38.5%; 95% CI, 36.4%-40.6%) received first-trimester ultrasonography. Compared with 1 308 879 general population pregnancies, the odds of antenatal care were lower for the first-trimester visit (odds ratios [ORs], 0.11 [95% CI, 0.09-0.13] in prison pregnancies and 0.23 [95% CI, 0.21-0.25] in prison control pregnancies), 8 or more antenatal care visits (ORs, 0.16 [95% CI, 0.14-0.19] in prison pregnancies and 0.25 [95% CI, 0.23-0.28] in prison control pregnancies), and first-trimester ultrasonography (ORs, 0.43 [95% CI, 0.36-0.50] in prison pregnancies and 0.51 [95% CI, 0.46-0.55] in prison control pregnancies).

**Conclusions and Relevance:**

This study found that women who experienced imprisonment were substantially less likely to receive adequate antenatal care than were women in the general population whether or not they were in prison during pregnancy. Efforts are needed to improve antenatal care for this population both in prison and in the community.

## Key Points

**Question:**

Do women who experience imprisonment in Ontario, Canada, have adequate antenatal care?

**Findings:**

In this cohort study of 626 pregnancies during time in prison and 2327 pregnancies in women with time in prison but not while pregnant, of the women who experienced imprisonment during pregnancy, 30.8% had a first-trimester visit, 48.4% had 8 or more antenatal care visits, and 34.5% received ultrasonography in their first trimester. Of the women who experienced imprisonment but not during pregnancy, 47.5% had a first trimester visit, 59.2% had 8 or more antenatal care visits, and 38.5% received ultrasonography in their first trimester.

**Meaning:**

The findings suggest that the odds of receiving antenatal care are lower for women who have experienced imprisonment than for the general population and that further research and interventions are needed to improve antenatal care in women who experience imprisonment.

## Teaser

his cohort study examines antenatal care quality indicators for women who experience imprisonment compared with women in the general population in Ontario, Canada.

## Introduction

Routine antenatal care is defined as “regular, standardized care that helps identify and treat complications and [promotes] health and wellbeing of the mother-baby unit in the pregnancy.”^1^ International, national, and regional guidelines define standards for routine antenatal care, which include access to first trimester ultrasonography to accurately determine the location of the fetus, number of fetuses, and gestational age; ultrasonography before 24 weeks’ gestation to detect fetal anomalies; timing and frequency of antenatal visits; and a minimum of 8 contacts with a health care professional, to reduce perinatal mortality and improve the woman’s experience of care.^1,2,3^ Antenatal care protects against prematurity, low birth weight, neonatal admission to a special care unit, and perinatal mortality.^4,5,6^ For women at increased risk of adverse perinatal outcomes, as suggested by previous preterm delivery or growth restriction, substance use, exposure to domestic violence, and certain medical and mental health conditions, the value of this care is heightened.^1^

Women who experience imprisonment tend to have poor health compared with the general population across a variety of health status indicators^7^ and are at higher risk of adverse pregnancy outcomes compared with the general population.^8,9,10,11^ That notwithstanding, data are lacking on antenatal care and challenges faced by pregnant women in prison.^12^ In a survey of wardens of 19 correctional facilities in the US, Ferszt et al^9^ found significant deficits in the care of pregnant women, including unmet nutritional needs, lack of access to primary care physicians, lack of access to lower bunks, and the routine use of restraints during transport and during labor. In 2014, Walker et al^8^ found that pregnant women in prison had higher odds of first accessing care late in pregnancy (ie, at or after 20 weeks’ gestation) compared with women in the community.

In the US, the National Commission on Correctional Health Care^13^ and the American Congress of Obstetrics and Gynaecology^14^ have established standards for pregnancy-related health care in correctional facilities. The standards are comparable to those for women in the community^15^ and include provisions to ensure the timely recognition of pregnancy,^13,14^ access to regular antenatal care and abortion services,^13,15^ and continuity of care after prison release.^14^ In addition, they state that women in prison should have access to appropriate screening and treatment for infectious diseases^13,16^ and mental health and substance-related disorders that are more common among people who experience incarceration^13,14,16^ and that prisons should provide prerelease and postrelease services for women reentering the community.^13^ In Canada, there are currently no analogous national standards for prisons and prison health care services, although jurisdictional or institutional policies may exist and health care professionals in prisons are obligated by their regulatory bodies to meet professional standards of care. In this study, we used population-based data to describe indicators of antenatal care quality, including antenatal care visits and completed ultrasonography, in women who experienced imprisonment and compared these data with data for the general population.

## Methods

### Study Design and Setting

We conducted a retrospective cohort study of pregnancies that led to in-hospital deliveries at 20 weeks’ gestation or greater. The study was population based because we included all eligible pregnancies in women in Ontario, Canada. The exposure was imprisonment status during pregnancy, and the outcomes were indicators of antenatal care quality, including antenatal care use and completed ultrasonography. This study was approved by the Hamilton Integrated Research Ethics Board. We did not obtain consent from participants because the data were deidentified, and this exemption was approved by the Hamilton Integrated Research Ethics Board. The study followed the Strengthening the Reporting of Observational Studies in Epidemiology (STROBE) reporting guideline.

In Ontario, provincial prisons are publicly funded and administered. They hold people who are admitted to custody without having been sentenced and who are sentenced to less than 2 years in custody. For Ontario residents, hospitalizations and medically necessary physician services are paid for through public health insurance, including in provincial prison. These services include antenatal care for women in provincial prison, which is provided through prison health care services or by referral to community-based care.

### Exposure

As described previously in a parallel article on infant and maternal outcomes of pregnancy,^11^ we used population-based health administrative data to identify singleton pregnancies with in-hospital deliveries at 20 weeks’ gestation or greater from January 1, 2005, to December 31, 2015, in women in Ontario. We included pregnancies from January 1, 2005, to December 31, 2015, because we had access to data on periods in provincial prison for those years for all women released from provincial prison in 2010; those data were obtained for an unrelated study.^17^ We used linked correctional data to identify pregnancies in women who had been released from provincial prison in Ontario in 2010. We called pregnancies in women who had not been released from a provincial prison in 2010 *general population pregnancies.* We stratified pregnancies in women released from provincial prison in 2010 based on whether the woman spent any time in prison during each pregnancy during the follow-up period (January 1, 2005, to December 31, 2015). This stratification allowed us to look at antenatal care first for women during the time in prison and immediately around imprisonment, given our interest in time around and during imprisonment as a challenging period in women’s lives, and in the prison setting as an opportunity to provide antenatal care and to link women with community-based health care. We expected that these 2 groups of women would have similar sociodemographic and morbidity characteristics. We called pregnancies in which women spent any time in prison during the pregnancy *prison pregnancies* and pregnancies in which women did not spend any time in prison during the pregnancy *prison control pregnancies*. Women who experienced imprisonment and who had multiple pregnancies during the follow-up period could contribute pregnancies to both the prison pregnancies and prison control pregnancies groups.

### Outcomes

We selected indicators of antenatal care based on international guidelines^2,3,18^ and available data in health administrative databases. We accessed insurance claims data from the Ontario Health Insurance Plan (OHIP), which pays for medically necessary physician services and tests for all Ontario residents, including those in provincial prison. We defined antenatal care as any visit to a family physician or obstetrician for which a specific billing or diagnostic code for pregnancy was used (eTable 1 in the Supplement). Because only 1 diagnostic code is used for ambulatory physician encounters in OHIP and physicians may have indicated a different diagnosis if they discussed multiple medical issues during an encounter, we also examined any visit to a family physician or obstetrician in pregnancy, which we called *ambulatory care visits in pregnancy*, as a more sensitive but less specific indicator of antenatal care. We examined any antenatal care and ambulatory care visits in the first trimester (ie, up to 12 weeks 6 days of gestation) and first-trimester ultrasonography (ie, 7 weeks’ 0 days’ and 12 weeks’ 6 days’ gestation), which is important for pregnancy dating and for diagnosing multiple gestations.^19^ We also examined whether women had 8 or more antenatal care and ambulatory care visits in pregnancy because 8 antenatal care visits is the minimum number recommended by the World Health Organization.^3^ Finally, we examined second-trimester ultrasonography, including any ultrasonography performed between 18 weeks’ 0 days’ and 22 weeks’ 6 days’ gestation, which is the recommended period for routine screening anatomy ultrasonography, and ultrasonography performed between 13 weeks’ 0 days’ and 27 weeks’ 6 days’ gestation; we included the longer period to capture cases still within a window that would likely identify major fetal (eg, major anomalies) or obstetrical (eg, placenta previa) complications commonly identified in the second trimester.^20^

### Covariates

We accessed correctional data on self-reported maternal race for prison pregnancies and prison control pregnancies, which was of interest given the overrepresentation of Black and Indigenous women in provincial prisons in Ontario and related health equity concerns. We combined several categories that included less than 5% of women into an *other* category. We used postal code data to derive neighborhood income quintile and rurality at the time of delivery for each pregnancy. At the time of each pregnancy, we accessed data on age, parity, specific medical diagnoses ever, and mental health diagnoses in the past 2 years using health administrative data.^11^

### Statistical Analysis

For each outcome, we fit regression models to compare the odds of each outcome between prison pregnancies and prison control pregnancies with general population pregnancies, and we also fit regression models to compare prison pregnancies with prison control pregnancies. We used logistic regression models with generalized estimating equations to account for correlation across pregnancies for each woman. We generated prevalence estimates and 95% CIs from generalized estimating equations postestimation commands.

For prison pregnancies, we described the amount of time spent in prison during the pregnancy. We calculated the number (percentage and 95% CIs) for pregnancy stage–specific outcomes for women who were in prison for 1 week or longer during that pregnancy stage, assuming that 1 week or longer would represent adequate time to provide a care visit or ultrasonography.

For the prison control pregnancies, we did not exclude pregnancies that occurred before first imprisonment because we were interested in risk for this group overall, not only after imprisonment. As such, we may have included pregnancies for some women who had not yet experienced imprisonment at the time of that pregnancy, for example, a woman with a pregnancy in 2007 who was first in prison between 2009 and 2010. Given the importance of identifying people who experience imprisonment clinically to explore and address any antenatal care needs and because physicians can only determine a woman’s experience with imprisonment *after* imprisonment, we conducted sensitivity analyses in which we included only deliveries after prison release in 2010. In these analyses, we could be certain that the prison controls group included only deliveries after a period of imprisonment.

 Statistical analyses were conducted between January 1, 2017, and May 4, 2020, using SAS Enterprise Guide, versions 6.1 and 7.1 (SAS Institute Inc).

## Results

**Figure 1.**
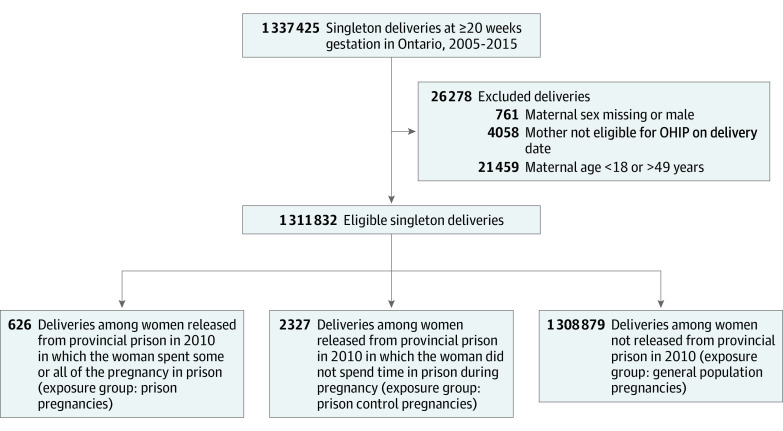
Flow Diagram for Identification of Eligible Deliveries and Assignment to Exposure Group OHIP indicates Ontario Health Insurance Plan.

We identified 626 prison pregnancies in 529 women, 2327 prison control pregnancies in 1570 women, and 1 308 879 general population pregnancies in 884 063 women (Figure 1). Characteristics of women at the time of delivery are given in Table 1. Mean (SD) age at the time of initial pregnancy during the follow-up period was 26.6 (5.4) years for prison pregnancies, 26.2 (5.4) years for prison control pregnancies, and 30.3 (5.3) years for general population pregnancies. Mean (SD) parity was 1.5 (1.5) for prison pregnancies and 1.4 (1.4) for prison control pregnancies compared with 0.7 (0.9) for general population pregnancies. We found that 301 prison pregnancies (48.1%) occurred in women with a mental health disorder based on health administrative data for the past 2 years compared with 670 prison control pregnancies (28.8%). Specifically, the prevalence of a substance use disorder was high in both these groups (247 [39.5%] in the prison pregnancy group and 471 [20.2%] in the prison control group).

**Table 1.** Characteristics of Women Who Experienced Imprisonment and Women in the General Population in Ontario at the Time of Delivery, by Pregnancies, From 2005 to 2015^a^

Characteristic	Prison pregnancies (n = 626)	Prison control pregnancies (n = 2327)	General population pregnancies (n = 1 308 879)
Age, y			
Mean (SD)	26.6 (5.4)	26.2 (5.4)	30.3 (5.3)
18-24	266 (42.5)	1035 (44.5)	190 635 (14.6)
25-34	299 (47.8)	1085 (46.6)	830 464 (63.4)
35-40	51 (8.1)	184 (7.9)	256 498 (19.6)
41-49	10 (1.6)	23 (1.0)	31 282 (2.4)
Race/ethnicity^b^			
Aboriginal	149 (23.8)	595 (25.6)	NR
Black	57 (9.1)	169 (7.3)	NR
White	345 (55.1)	1163 (50.0)	NR
Other	36 (5.8)	139 (6.0)	NR
Missing	39 (6.2)	261 (11.2)	NR
Rural residence			
No	515 (82.3)	1838 (79.0)	1 177 201 (89.9)
Yes	106 (16.9)	489 (21.0)	131 594 (10.1)
Missing	≤5 (0.8)^c^	0 (0.0)	84 (0.0)
Neighborhood income, quintile			
1, lowest	273 (43.6)	1026 (44.1)	287 097 (21.9)
2	134 (21.4)	473 (20.3)	261 149 (20.0)
3	80 (12.8)	333 (14.3)	266 035 (20.3)
4	61 (9.7)	208 (8.9)	275 260 (21.0)
5	47 (7.5)	193 (8.3)	213 348 (16.3)
Missing	31 (5.0)	94 (4.0)	5990 (0.5)
Parity			
Mean (SD)	1.5 (1.5)	1.4 (1.4)	0.7 (0.9)
Median (IQR)	1 (0-2)	1 (0-2)	1 (0-1)
Medical diagnosis			
Diabetes	9 (1.4)	50 (2.1)	26 807 (2.0)
Hypertension	6 (1.0)	21 (0.9)	33 304 (2.5)
Viral hepatitis	69 (11.0)	149 (6.4)	24 734 (1.9)
HIV infection	≤5 (0.6)^c^	18 (0.8)	804 (0.1)
Mental disorder diagnosis			
Any	301 (48.1)	670 (28.8)	32 322 (2.5)
Substance-related disorders	247 (39.5)	471 (20.2)	8425 (0.6)
Anxiety disorders	104 (16.6)	261 (11.2)	19 622 (1.5)
Mood disorders	83 (13.3)	195 (8.4)	10 134 (0.8)
Schizophrenia	32 (5.1)	54 (2.3)	1233 (0.1)
Self-harm	57 (9.1)	124 (5.3)	3849 (0.3)

Abbreviations: IQR, interquartile range; NR, not reported.

^a^ Data are presented as number (percentage) of pregnancies unless otherwise indicated; some women had multiple pregnancies during the follow-up period. All eligible pregnancies in Ontario from January 1, 2005, to December 31, 2015, were included. *Prison pregnancies* were eligible pregnancies in women released from provincial prison in 2010 and during which the woman spent any time in prison. *Prison control pregnancies* were eligible pregnancies in women released from provincial prison in 2010 and during which women did not spend any time in prison.

^b^ Data on race/ethnicity were available for prison pregnancies and prison control pregnancies only.

^c^ The true value is between 1 and 5 and was suppressed, as per ICES policy. We used the maximum value of 5 to calculate the prevalence.

**Figure 2.**
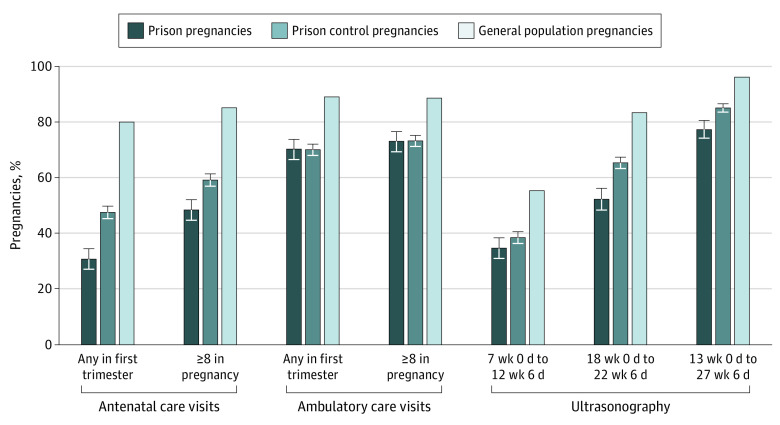
Percentage of Pregnancies That Met Antenatal Care Quality Indicators for Women Who Experienced Imprisonment and in the General Population in Ontario, Canada, 2005 to 2015 All eligible pregnancies in Ontario between 2005 and 2015 were included. *Prison pregnancies* were eligible pregnancies in women released from provincial prison in 2010 and during which the woman spent any time in prison. *Prison control pregnancies* were eligible pregnancies in women released from provincial prison in 2010 and during which women did not spend any time in prison. Data are presented for women by pregnancy, and some women had multiple pregnancies during the follow-up period. Error bars indicate 95% CIs.

In prison pregnancies, 193 women (30.8%) had a first-trimester visit, 272 (48.4%) had 8 or more antenatal visits during pregnancy, 209 (34.6%) underwent ultrasonography between 7 weeks 0 days and 12 weeks 6 days, and 320 (52.3%) underwent ultrasonography between 18 weeks 0 days and 22 weeks 6 days (Table 2 and Figure 2). In prison control pregnancies, 1356 (47.5%) had a first-trimester visit, 1106 (59.2%) had 8 or more antenatal care visits during pregnancy, 893 (38.5%) had first-trimester ultrasonography, and 1512 (65.4%) had ultrasonography between 18 weeks 0 days and 22 weeks 6 days.

**Table 2.** Antenatal Care Quality Indicators in Pregnancies in Women Who Experienced Imprisonment and in the General Population in Ontario, From 2005 to 2015^a^

Antenatal care indicator	Prison pregnancies (n = 626)		Prison control pregnancies (n = 2327)		General population pregnancies (n = 1 308 879)	
	No.	Frequency, % (95% CI)^b^	No.	Frequency, % (95% CI)^b^	No.	Frequency, % (95% CI)^b^
**Antenatal care visits**						
Any first trimester	193	30.8 (27.1-34.6)	1106	47.5 (45.3-49.8)	1 045 526	79.9 (79.8-80.0)
≥8 in pregnancy	272	48.4 (44.4-52.4)	1356	59.2 (56.9-61.4)	1 111 274	85.1 (85.1-85.2)
**Ambulatory care visits**						
Any first trimester	425	70.3 (66.5-73.8)	1612	70.1 (68.0-72.1)	1 164 186	89.1 (89.0-89.1)
≥8 in pregnancy	427	73.1 (69.3-76.5)	1678	73.3 (71.2-75.2)	1 156 794	88.6 (88.6-88.7)
**Ultrasonography**						
7 wk 0 d to 12 wk 6 d	209	34.6 (31.0-38.4)	893	38.5 (36.4-40.6)	721 889	55.3 (55.2-55.3)
18 wk 0 d to 22 wk 6 d	320	52.3 (48.3-56.2)	1512	65.4 (63.3-67.4)	1 092 059	83.4 (83.4-83.5)
13 wk 0 d to 27 wk 6 d	477	77.5 (74.0-80.6)	1971	85.1 (83.4-86.6)	1 259 287	96.2 (96.1-96.2)

^a^ Data are presented for women by pregnancy; some women had multiple pregnancies during the follow-up period. All eligible pregnancies in Ontario between 2005 and 2015 were included. *Prison pregnancies* were eligible pregnancies in women released from provincial prison in 2010 and during which the woman spent any time in prison. *Prison control pregnancies* were eligible pregnancies in women released from provincial prison in 2010 and during which women did not spend any time in prison.

^b^ Generated with postestimation commands to account for correlation with multiple pregnancies.

Compared with general population pregnancies, women with prison pregnancies had significantly lower odds of a first-trimester visit (odds ratio [OR], 0.11; 95% CI, 0.09-0.13), 8 or more antenatal visits during pregnancy (OR, 0.16; 95% CI, 0.14-0.19), and ultrasonography between 7 weeks 0 days and 12 weeks 6 days (OR, 0.43; 95% CI, 0.36-0.50) (Table 3). Similarly, women with prison control pregnancies had significantly lower odds of first-trimester visit (OR, 0.23; 95% CI, 0.21-0.25), 8 or more antenatal visits during pregnancy (OR, 0.25; 95% CI, 0.23-0.28), and ultrasonography between 7 weeks 0 days and 12 weeks 6 days (OR, 0.51; 95% CI, 0.46-0.55). Comparing prison pregnancies with prison control pregnancies, women had significantly lower odds of a first-trimester antenatal care visit (OR, 0.53; 95% CI, 0.44-0.64), 8 or more antenatal care visits in pregnancy (OR, 0.61; 95% CI, 0.52-0.73), and ultrasonography between 7 weeks 0 days and 12 weeks 6 days (OR, 0.83; 95% CI, 0.70-0.99) but no difference in any first-trimester ambulatory care visit (OR, 1.00; 95% CI, 0.83-1.21) or 8 or more ambulatory care visits in pregnancy (OR, 0.95; 95% CI, 0.78-1.15).

**Table 3.** Unadjusted ORs for Antenatal Care Quality Indicators in Pregnancies in Women Who Experienced Imprisonment and in the General Population in Ontario, 2005-2015^a^

Antenatal care indicator	Unadjusted OR (95% CI)^b^		
	Prison pregnancies vs general population pregnancies	Prison control pregnancies vs general population pregnancies	Prison pregnancies vs prison control pregnancies
**Antenatal care visits**			
Any first trimester	0.11 (0.09-0.13)	0.23 (0.21-0.25)	0.53 (0.44-0.64)
≥8 in pregnancy	0.16 (0.14-0.19)	0.25 (0.23-0.28)	0.61 (0.52-0.73)
**Ambulatory care visits**			
Any first trimester	0.29 (0.24-0.35)	0.29 (0.26-0.32)	1.00 (0.83-1.21)
≥8 in pregnancy	0.35 (0.29-0.42)	0.35 (0.32-0.39)	0.95 (0.78-1.15)
**Ultrasonography**			
7 wk 0 d to 12 wk 6 d	0.43 (0.36-0.50)	0.51 (0.46-0.55)	0.83 (0.70-0.99)
18 wk 0 d to 22 wk 6 d	0.22 (0.19-0.26)	0.37 (0.34-0.41)	0.59 (0.50-0.71)
13 wk 0 d to 27 wk 6 d	0.14 (0.11-0.17)	0.23 (0.20-0.26)	0.62 (0.50-0.78)

Abbreviation: OR, odd ratio.

^a^ Data are presented for women by pregnancy; some women had multiple pregnancies over the follow-up period. All eligible pregnancies in Ontario between 2005 and 2015 were included. *Prison pregnancies* were eligible pregnancies in women released from provincial prison in 2010 and during which the woman spent any time in prison. *Prison control pregnancies* were eligible pregnancies in women released from provincial prison in 2010 and during which women did not spend any time in prison.

^b^ Generated with postestimation commands to account for correlation with multiple pregnancies.

When we limited pregnancies in the prison control pregnancies to those after release from provincial prison in 2010, there was no substantial change in antenatal care quality indicators (eTable 2 in the Supplement).

For the 626 prison pregnancies, 291 women (46.5%) were in prison for 15 days or less during the pregnancy, 87 (13.9%) were in prison for 16 to 30 days, and 248 (39.6%) were in prison for more than 30 days.

 For women who spent 1 week or longer in provincial prison in their first trimester, 85 (30.7%) had a first trimester antenatal care visit, and for women who spent 1 week or longer in prison between 7 weeks 0 days and 12 weeks 6 days gestation, 113 (39.6%) received first trimester ultrasonography. A total of 28 women (38.3%) who spent 1 week or longer in prison between 18 weeks 0 days and 22 weeks 6 days of gestation underwent ultrasonography during that period (eTable 3 in the Supplement).

## Discussion

In this population-based study, we found that women who experienced imprisonment were less likely to receive antenatal care across all indicators studied than were women in the general population whether or not they were in prison during pregnancy. Women who were in prison during pregnancy stages when an antenatal visit or ultrasonography was indicated often did not receive this indicated care. Because most women were in prison during pregnancy for only a few weeks during prison pregnancies and, by definition, women were not in prison at all during prison control pregnancies, these data indicate a lack of antenatal care for this population in the community before incarceration and after prison release as well as in prison.

Although there is a paucity of research on antenatal care in women who experience imprisonment, our findings are consistent with those from Walker et al^8^ regarding a lack of early antenatal care in women in prison during pregnancy compared with women in the general population and regarding inadequate antenatal care in other populations that are overrepresented in prisons, such as women with HIV infection and women with serious mental illness.^21,22^ Although not directly comparable, our data on ultrasonography for the general population are consistent with data from another study,^23^ suggesting internal validity. Prior research indicates that people have higher mean ambulatory care use rates while in prison compared with their use after release.^17^ Although we did not specifically calculate rates of antenatal care use per time in prison and in the community, our findings suggest that women with time in prison during pregnancy are less likely to receive antenatal care than are women with experience of imprisonment but not during pregnancy, which is inconsistent with the prior research on ambulatory care use for all women who experienced imprisonment. There may be qualitatively different issues associated with overall health care access and use or, specifically, antenatal care access and use for women who experience imprisonment while pregnant compared with other people who experience imprisonment, and this issue deserves further attention.

There are likely many factors associated with the between-group differences that we identified in this study. Compared with women in the general population, women who experience imprisonment typically have greater morbidity^7^ and greater social challenges. Although morbidity and behaviors, such as use of substances, would increase the importance of antenatal care, women who experience imprisonment may have priorities that compete with antenatal care use and may face structural barriers to accessing care. These factors would differ while in prison and in the community: in the community, housing, family responsibilities, and substance use may play a bigger role, and in prison, women may need to deal with medical issues, such as withdrawal, and logistical issues, such as court attendance and challenges in accessing health care.^24^ In addition, the time around imprisonment may represent a period of increased challenges for women who experience imprisonment, for example, related to illicit drug use, which may lead to imprisonment itself in the context of criminalization and could also be associated with less antenatal care. This issue could further explain the difference between prison pregnancies and prison control pregnancies (ie, worse antenatal care use for women during prison pregnancies may be associated in part with a challenging period in women’s lives as well as with imprisonment itself).

In the context of universal health care in Ontario, the finding that women who experienced imprisonment had worse antenatal care compared with other women is troubling. Putatively, this inequity may be even greater in the absence of a universal health care system, such as in the US, given the overrepresentation of women with worse socioeconomic status in prison populations. Given the potential sequelae of poor antenatal care and considering the high prevalence of risk factors for adverse maternal and infant outcomes in this population, we are concerned about unmet health care needs that may lead to harms for parent and child and may exacerbate existing health disparities for this population. Interventions are necessary to support antenatal care for this population in both prison and the community.^12,25,26^ Potential opportunities to improve reproductive health in this population include enhanced health care and programs for women in prison, interventions to link women leaving prison with high-quality primary care and antenatal care in the community, access to care and education to prevent unintended pregnancy, and policy changes to reduce the number of women who experience imprisonment.^27^

Health administrative data allowed us to examine whether and when women obtained antenatal care but do not provide detailed information regarding the quality or acceptability of health care that they received or that is available. We recommend research to define barriers to antenatal care, including related to accessibility and acceptability of services in prison and in the community, competing priorities for women who experience imprisonment while in prison and in the community, and awareness by women who experience imprisonment and health care professionals of recommendations for pregnancy screening and routine antenatal care in prison. In particular, qualitative data from the perspective of women with lived experience of pregnancy and imprisonment would be valuable. In addition, future research should examine antenatal care access and quality in all pregnancies, including those that end in spontaneous or induced abortion before 20 weeks’ gestation.

### Strengths and Limitations

Strengths of this study are that we used population-based data for a large sample of women who experienced imprisonment, examined a variety of antenatal care quality indicators, and compared data for women who experience imprisonment and women in the general population.

The study also has limitations. We included only women with pregnancies that led to delivery at 20 weeks’ gestation or greater given our interest in antenatal care as a contributor to both maternal and child health. Of note, antenatal care in these pregnancies may be systematically different than antenatal care in pregnancies that ended in spontaneous or induced abortion before 20 weeks’ gestation. For example, women who terminated their pregnancies may have been more likely to obtain first-trimester care and ultrasonography, which would be required before a termination. We note the substantial difference in antenatal care compared with ambulatory care use in women with prison pregnancies. This finding likely reflects that women who experienced imprisonment had high morbidity and high rates of ambulatory care use in prison and in the community^28^ and may also result from the limit of 1 diagnostic code per outpatient visit in OHIP because some women may have had visits that addressed multiple medical issues, including pregnancy. However, using either definition, care use did not meet the standards for antenatal care and was significantly worse for women who experienced imprisonment compared with the general population. Although the population of women who experience imprisonment is heterogeneous in terms of various sociodemographic and morbidity factors, we did not examine antenatal care quality indicators for subgroups of the population, for example, for women with substance use disorders. This type of stratified analysis would provide a more nuanced understanding of antenatal care within this population and could inform the appropriate targeting and tailoring of interventions for specific subgroups of women. This study is descriptive rather than explanatory, and consistent with our objectives, we did not attempt to control for potentially confounding variables^29^; specifically, we did not attempt to define a causal effect of imprisonment on antenatal care quality indicators.

## Conclusions

This cohort study found that pregnant women in Ontario who experienced imprisonment were significantly less likely to receive adequate antenatal care than other women in the general population. Given that poor antenatal care is associated with risks to maternal and child health and the high prevalence of risk factors for adverse maternal and neonatal outcomes in this population, work appears to be urgently required to support high-quality antenatal care for women who experience imprisonment.

## Notes

Supplement. **eTable 1.** Codes for Outcomes **eTable 2.** Antenatal Care Quality Indicators in Prison Control Pregnancies, for All Pregnancies 2005 to 2015 and for Pregnancies 2010 to 2015 **eTable 3.** Antenatal Care Quality Indicators by Timing and Length of Time in Prison During Pregnancies Between 2005 and 2015 in Women in Ontario Who Experienced Imprisonment and Were in Prison for Part of Their Pregnancy, N=626
