# Supplementary material for: Quality of Antenatal Care for Women Who Experience Imprisonment in Ontario, Canada
Source: JAMA Netw Open. 2020 Aug 6;3(8):e2012576. doi: 10.1001/jamanetworkopen.2020.12576 (PMC7411537; doi:10.1001/jamanetworkopen.2020.12576)
Supplement: Supplement. — eTable 1. Codes for Outcomes eTable 2. Antenatal Care Quality Indicators in Prison Control Pregnancies, for All Pregnancies 2005 to 2015 and for Pregnancies 2010 to 2015 eTable 3. Antenatal Care Quality Indicators by Timing and Length of Time in Prison During Pregnancies Between 2005 and 2015 in Women in Ontario Who Experienced Imprisonment and Were in Prison for Part of Their Pregnancy, N=626 [file jamanetwopen-3-e2012576-s001.pdf]

# Supplementary Online Content

Carter Ramirez A, Liauw J, Cavanagh A, et al. Quality of antenatal care for women who experience imprisonment in Ontario, Canada. *JAMA Netw Open*. 2020;3(8):e2012576. doi10.1001/jamanetworkopen.2020.12576

**eTable 1.** Codes for Outcomes

**eTable 2.** Antenatal Care Quality Indicators in Prison Control Pregnancies, for All Pregnancies 2005 to 2015 and for Pregnancies 2010 to 2015

**eTable 3.** Antenatal Care Quality Indicators by Timing and Length of Time in Prison During Pregnancies Between 2005 and 2015 in Women in Ontario Who Experienced Imprisonment and Were in Prison for Part of Their Pregnancy, N=626

**This supplementary material has been provided by the authors to give readers additional information about their work.**

© 2020 Carter Ramirez A et al. *JAMA Network Open.*

**eTable 1.** Codes for Outcomes

| Definition | OHIP codes |
|---|---|
| Antenatal care | P003, P004, or P005; OR<br>A code and diagnosis code= 632-677 or 970; OR<br>K code and diagnosis code= 632-677 or 970 |
| Ambulatory care | Family Physician and Obstetrician billings with codes A, K, P003, P004, or P005 |
| Ultrasound | J157-J160, J457-J460 |

**eTable 2.** Antenatal Care Quality Indicators in Prison Control Pregnancies,[a] for All Pregnancies 2005 to 2015 and for Pregnancies 2010 to 2015

| Antenatal care indicator | | Prison control pregnancies 2005 to 2015, N=2,327 | | Prison control pregnancies 2010 to 2015, N=1,143 | |
|---|---|---|---|---|---|
| | | n | % (95% CI)[b] | n | % (95% CI)[b] |
| Antenatal care visits | Any first trimester | 1,106 | 47.5 (45.3-49.8) | 584 | 51.2 (48.1-54.3) |
| | ≥8 in pregnancy | 1,356 | 59.2 (56.9-61.4) | 703 | 61.4 (58.3-64.5) |
| Ambulatory care visits | Any first trimester | 1,612 | 70.1 (68.0-72.1) | 809 | 71.46 (68.6-74.2) |
| | ≥8 in pregnancy | 1,678 | 73.3 (71.2-75.2) | 872 | 76.78 (74.1-79.3) |
| Ultrasound | 7 wk 0 d to 12 wk 6 d | 893 | 38.5 (36.4-40.6) | 463 | 40.6 (37.7-43.5) |
| | 18 wk 0 d to 22 wk 6 d | 1,512 | 65.4 (63.3-67.4) | 817 | 71.6 (68.8-74.3) |
| | 13 wk 0 d to 27 wk 6 d | 1,971 | 85.1 (83.4-86.6) | 1,015 | 89.0 (86.9-90.8) |

[a]Prison control pregnancies were eligible pregnancies in women released from provincial prison in 2010 and in which women did not spend any time in prison during pregnancy. Data are presented for women by pregnancy, and some women had multiple pregnancies over the follow up period. [b]Generated with post-estimation commands to account for correlation between multiple pregnancies.

**eTable 3.** Antenatal Care Quality Indicators by Timing and Length of Time in Prison During Pregnancies Between 2005 and 2015 in Women in Ontario Who Experienced Imprisonment and Were in Prison for Part of Their Pregnancy,[a] N=626

| Antenatal care quality indicator | Prison exposure | n | % (95% CI) |
|---|---|---|---|
| Any first trimester antenatal care visit | ≥1 week in prison first trimester | 85 | 30.7 (25.8-36.1) |
| Any first trimester ambulatory care visit | ≥1 week in prison first trimester | 233 | 80.3 (75.1-84.7) |
| Ultrasound 7+0 to 12+6 weeks | ≥1 week in prison 7+0 to 12+6 | 113 | 39.6 (34.1-45.4) |
| Ultrasound 18+0 to 22+6 weeks | ≥1 week in prison 18+0 to 22+6 | 28 | 38.3 (28.3-49.5) |
| Ultrasound 13+0 to 27+6 weeks | ≥1 week in prison 13+0 to 27+6 | 144 | 73.1 (66.4-78.9) |

[a]Of all pregnancies in Ontario between 2005 and 2015, prison pregnancies were eligible pregnancies in women released from provincial prison in 2010 and in which the woman spent any time in prison. Data are presented for women by pregnancy, and some women had multiple pregnancies over the follow up period.

© 2020 Carter Ramirez A et al. *JAMA Network Open.*
